# Novel analytical method for detection of orange juice adulteration based on ultra-fast gas chromatography

**DOI:** 10.1007/s00706-018-2233-8

**Published:** 2018-08-09

**Authors:** Anna Różańska, Tomasz Dymerski, Jacek Namieśnik

**Affiliations:** Department of Analytical Chemistry, Faculty of Chemistry, Gdańsk University of Technology, Narutowicza 11/12, 80-233 Gdańsk, Poland

**Keywords:** Clusters, Electronic nose, Fruit juices, Gas chromatography, Random Forest

## Abstract

**Abstract:**

The food authenticity assessment is an increasingly important issue in food quality and safety. The application of an electronic nose based on ultra-fast gas chromatography technique enables rapid analysis of the volatile compounds from food samples. Due to the fact that this technique provides chemical profiling of natural products, it can be a powerful tool for authentication in combination with chemometrics. In this article, a methodology for classification of Not From Concentrate (NFC) juices was presented. During research samples of 100% orange juice, 100% apple juice, as well as mixtures of these juices with known percentage of base juices were tested. Classification of juice samples was carried out using unsupervised and supervised statistical methods. As chemometric methods, Hierarchical Cluster Analysis, Classification Tree, Naïve Bayes, Neural Network, and Random Forest classifiers were used. The ultra-fast GC technique coupled with supervised statistical methods allowed to distinguish juice samples containing only 1.0% of impurities. The developed methodology is a promising analytical tool to ensure the authenticity and good quality of juices.

**Graphical abstract:**

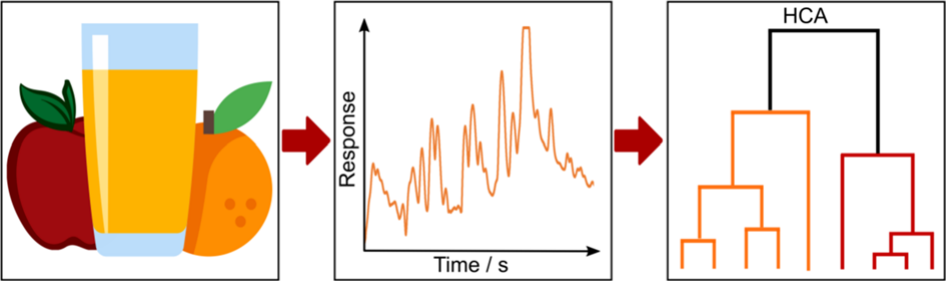

## Introduction

A juice manufacturing is one of the major branches which can be distinguished in the food industry. Due to the fact that the production of the fruit is seasonal, the fruit juice manufacturing allows us to consume them during the whole year. The most commonly consumed one is orange juice [1]. Its production accounts for nearly 85% of total citrus juice consumption [2].

Food quality assessment is increasingly important in the food industry. There are many types of fruit juice adulterations. The main one is dilution with water, which reduces the content of soluble solids, such as sugars or organic acids [3] or fragrance extracts and colourants [4]. Moreover, very popular type of fruit juice sophistication is the addition of cheaper fruit juices [3]. Orange juices are most often adulterated with the addition of mandarin [5, 6], tangerine [7], lemon [8], or grapefruit [9] juices.

In recent years, the interest in the healthy and balanced diet is growing. The consumption of orange juice allows not only to supply nutrients, but it can also have a positive effect on the human organism. Wabner et al. proved that orange juice improved blood lipid profiles in subjects with moderate hypercholesterolemia [10]. Furthermore, orange juice intake with the high-fat, high-carbohydrate meal prevented meal-induced oxidative and inflammatory stress, and moreover, it prevents the expression of plasma endotoxin and Toll-like receptors [11]. The consumption of this type of fruit juice is beneficial in the control of calcareous and uric acid nephrolithiasis [12].

One of the healthiest juices is raw, and naturally, cloudy Not From Concentrate (NFC) juice, due to their composition, is most similar to the composition of fruits from which they are obtained. According to the European Fruit Juice Association, over the past 5 years, the demand for NFC juices has increased. Across Europe, the increase was of about 14.0%. Moreover, in Poland, consumption of NFC juices increased nearly tenfold [13]. Such an intense increase in the demand for juices can cause a decrease in the product quality. According to experts, orange juice in Poland can be diluted by the addition of apple juice, which is cheaper and more easily accessible.

There are many reference methods to assess the quality of juices. Among them, chemical, physical, and microbiological methods can be mentioned. Orange juice is a widespread subject of research regarding the analysis of the aroma profile and monitoring of processes occurring in fruit juices [14, 15]. Samples of these juices are also classified into NFC, From Concentrate (FC), and pasteurized juices using chromatographic techniques [16–18] and e-nose devices [19, 20]. For detecting adulterations of orange juices, the most effective are methods in which spectrometry and chromatographic techniques are involved [21, 22]. However, these procedures are time-consuming, labour-intensive, or expensive. For this reason, new solutions that allow for a rapid assessment of the quality of fruit juices are sought. Devices enable rapid analysis are called electronic noses [23, 24]. The electronic nose is a device which makes possible to detect and distinguish complex mixtures of fragrances. The advantage of this equipment guarantees low time consumption and low costs of single analysis, the omission of sample preparation step and the possibility of in situ measurement. The applications of e-nose to analyze aroma of food products are shown in a number of reports [25–30]. An electronic nose is a useful tool for classification fruit juice samples [31–33].

There is a lack of literature reports about the research of adulteration of orange juice by apple juice addition. This also includes electronic nose investigations. Therefore, the aim of this study was to develop a methodology for rapid evaluation of the authenticity of orange juices. For this purpose, the aroma profiles of orange juice, apple juice, and mixtures of both juices were compared by the use of e-nose based on ultra-fast gas chromatography. Moreover, e-nose analyses were combined with chemometric methods. Provided investigations can be supplementary to other control methods used for fruit-juice quality assessment.

## Results and discussion

During the process of food quality control, from several dozens to several hundred samples need to be analyzed. For this reason, much less time-consuming methods are sought. In this work, the ultra-fast gas chromatography technique was used. Duration of the measurement was less than 2 min. Regarding that fact, chromatographic separation may be insufficient. This is particularly problematic when samples with a very complex matrix composition are subjected to testing. In the research, a holistic approach was used. This approach uses the fingerprint method, i.e., the entire chromatograms of the samples are compared using statistical data analysis.

**Fig. 1:**
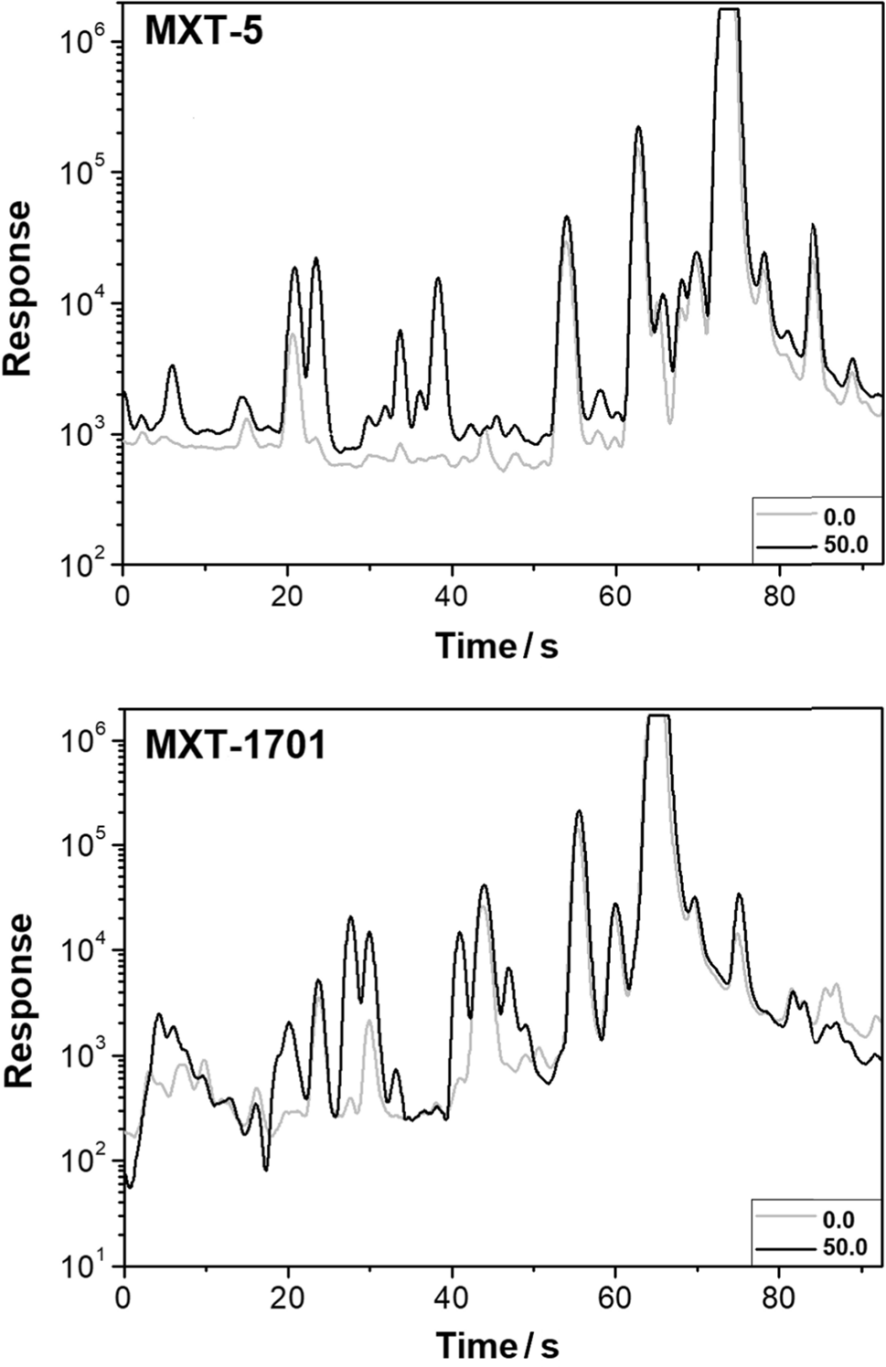
Chromatographic fingerprints for 100% orange juice (0.0) and a mixture of 50.0% orange juice and 50.0% apple juice (50.0)

Figure 1 shows the fingerprints obtained for samples of 100.0% orange juice (0.0) and a mixture of 50.0% orange juice and 50.0% apple juice (50.0) for both chromatographic columns (MXT-5 and MXT-1701). The fingerprints show the differences in the composition of the headspace of unadulterated orange juice and orange juice with the addition of apple juice. As it can be seen in Fig. 1, the signals corresponding to the chemical compounds detected in the samples of the juice mixture (50.0) are much more intense compared to the samples of orange juice (0.0). Furthermore, as a result of the addition of cheaper juice, it can be observed more signals in the fingerprint. Peaks detected in adulterated juice may be characteristic of apple juice. Identification of these chemical compounds is very important due to the fact that these compounds may be potential markers of adulteration of orange juice with apple juice. Moreover, the determination of these markers in volatile fraction allows designating the quality of orange juice.

After chromatographic measurements, chemometric analyses were performed. The chromatographic peak areas corresponding to detected chemical compounds were used as an input data. All tested samples were compared based on the similarities and differences in the composition of the volatile fraction. During data processing with a large number of variables, there is a high probability of “voodoo correlation” [34]. As a general rule, to avoid this type of accidental correlation, the number of measurements should be fivefold greater than the number of variables. In the presented studies, over 120 chemical compounds (variables) were detected during each analysis. However, carrying out over 600 analyses is not easy to realize. Therefore, instead of increasing the number of measurements, it was decided to reduce the number of variables to 10.

Table 1 shows selected ten chemical compounds on the basis of the analysis of variance ANOVA. These compounds showed the greatest relative changes in concentration during testing and, therefore, had the greatest impact on the result of statistical analysis. It should be noted that these compounds are not necessarily those that have the highest concentrations in the volatile fraction of the sample, but these are the compounds for which the respective chromatographic peak areas show the greatest deviations depending on the percentage of apple juice. These compounds can be considered as potential indicators of the quality of orange juice. The Kovats retention indexes for both chromatographic columns (KI—MXT-5, KII—MXT-1701) were given for each potential quality markers of orange juice. In Table 1, aroma descriptors, which can be caused by the presence of selected chemical substances, are also listed. The six pre-selected chemical compounds listed in Table 1 (2-methylbutanol, ethyl butyrate, butyl acetate, 2-hexenal, 3-hexenol, and propan-2-one) are volatile organic compounds (VOCs) that have been identified in the volatile fraction of apples by Vrhovsek et al. and Mattheis et al. [35, 36]. The main chemical compounds affecting the aromas of these fruits are esters, mainly ethyl butyrate and butyl acetate, as well as alcohols, among others 2-methylbutanol and six-carbon compounds, such as 2-hexenol, which have been identified in various apple varieties: Redchief, Granny, or Golden [37, 38]. However, different varieties of apples are characterized by different compositions of their volatile fractions. For example, propan-2-one is the substance, which was detected only in the headspace of the Bisbee Delicious apple samples [35].

**Table 1:** Selected compounds identified as potential orange juice quality markers

No.	Chemical compound	Kovats index		Aroma descriptors	Molar mass
		MXT-5	MXT-1701		
1	Propenal	450	566	Apple, fruity, sweet	56
2	2-Hexenal	854	956	Apple, cherry, fruity, green, strawberry	98
3	Butyl acetate	810	879	Banana, fruity, green, pear, pineapple, sweet	116
4	3-Hexenol	852	960	Fresh, green, leafy	100
5	Ethyl butyrate	799	864	Banana, fruity, pineapple, strawberry, sweet	116
6	2-Butanol	594	699	Alcoholic, winey	74
7	2-Methylbutanol	740	852	Fruity	88
8	*m*-Xylene	870	922	Plastic	106
9	Propan-2-one	478	586	Fruity	58
10	Methyl acetate	489	596	Blackcurrant, fruity	74

During the research, Hierarchical Cluster Analysis (HCA) was used as a chemometric model. HCA is a method that allows sorting data and binding them into natural groups based on their similarity [39]. At the beginning of the agglomeration procedure, each analyzed object is located in a separate cluster. Next, the number of clusters decreases in every step until the moment when all input data will belong to one cluster [40]. To group objects into clusters, it is necessary to define the numerical value of the similarity between objects. Usually, the Euclidean distance is used for this purpose [41]. However, in the presented research, the Ward method was used. This method is characterized by the fact that analysis of variance ANOVA is used to assess the distance between clusters [40, 42]. The application of this method allows obtaining the best results if the clusters are of equal size [41].

**Fig. 2:**
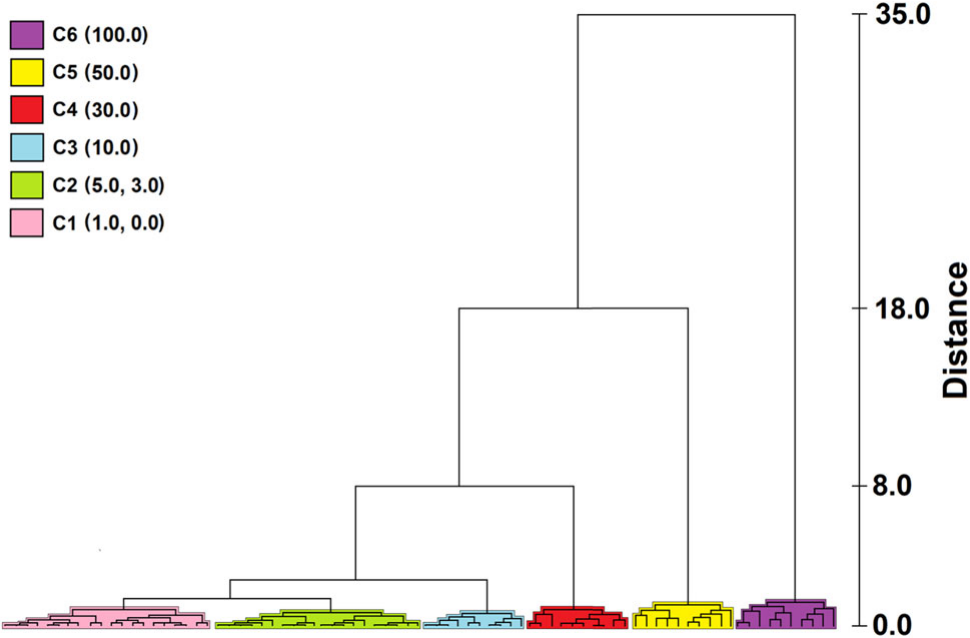
Classification of orange juices according to the percentage of apple juice content using HCA method

The purpose of the statistical analysis was a verification whether, using the proposed analytical procedure, it is possible to classify samples of unadulterated orange juice and samples of adulterated juice. Figure 2 shows the results of HCA. The composition of the aroma for 100.0% apple juice samples (marked as 100.0) forming a single cluster—C6. Subsequently, separated clusters (C5, C4, and C3) were obtained for samples of mixtures of orange and apple juice containing, respectively, 50.0, 30.0, and 10.0% of apple juice (marks as 50.0, 30.0, and 10.0). This means that the composition of the volatiles for these samples is statistically different and distinguishing them is not a problem. However, for data for samples containing from 1.0 to 5.0% of apple juice and samples of 100% orange juice (0.0), the distinction is difficult. The data create two clusters: one for the samples marked as 1.0 and 0.0 (C1) and the other for the samples marked as 3.0 and 5.0 (C2). This means that if the juice samples contain only 1% apple juice, this has a slight effect on the composition of the orange juice volatile fraction and these samples are classified as unadulterated samples. Whereas, samples marked as 3.0 and 5.0 are classified as samples of adulterated juice, but their aromas are so similar that it is difficult to distinguish them from each other.

**Fig. 3:**
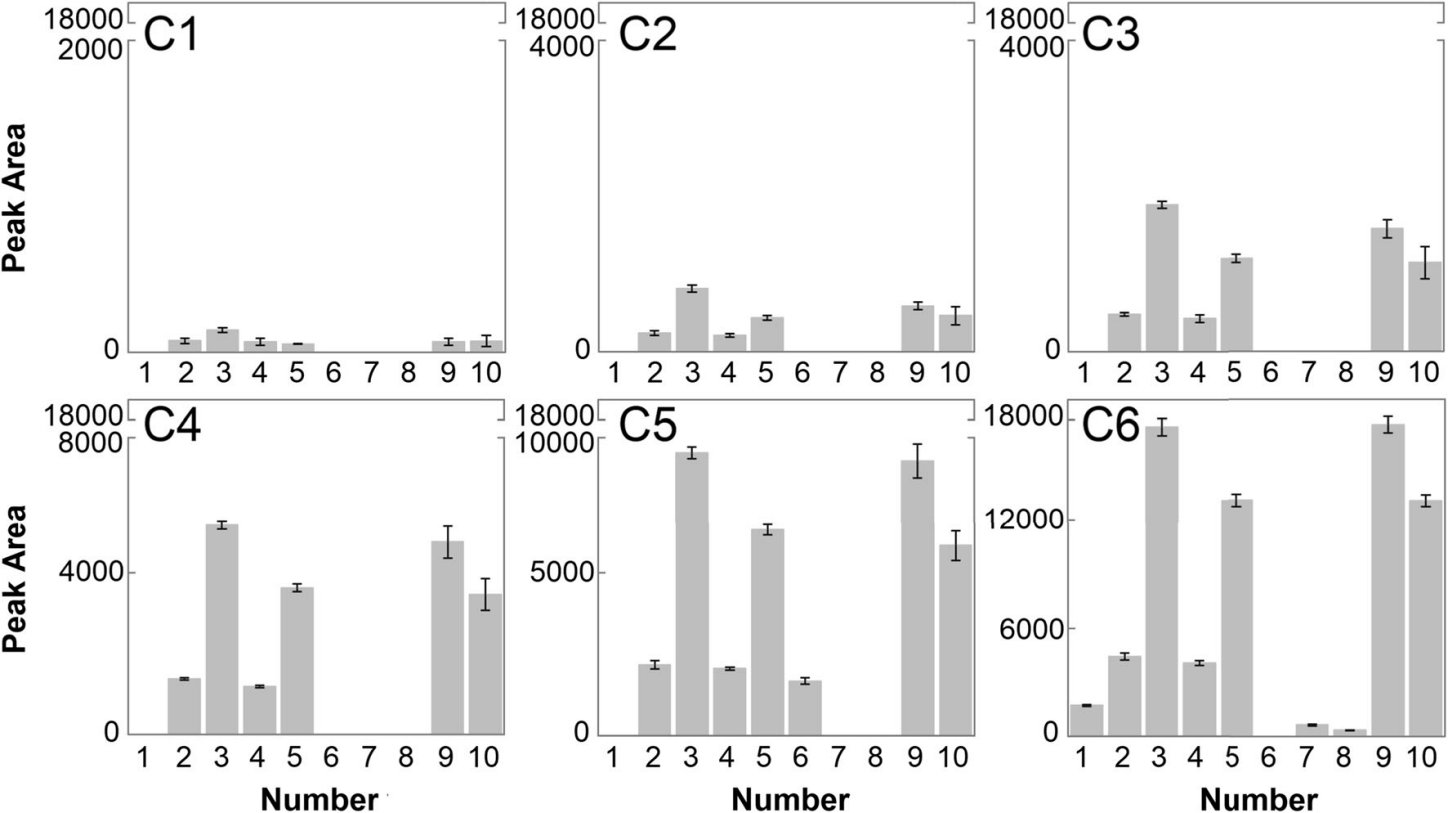
Histograms depicting the mean values with a standard deviation of chromatographic peak areas for selected chemical compounds (numbers correspond to Table 1) belonging to the six clusters, as illustrated in Fig. 2

Figure 3 shows the average of chromatographic peak areas for individual six clusters corresponding to the chemical compounds, as listed in Table 1. In the case of the first four clusters, six of ten selected substances were detected, namely: 2-hexenal, butyl acetate, 3-hexenol, ethyl butyrate, propan-2-one, and methyl acetate. The four remaining chemicals were below the limit of detection. In the samples assigned to the first cluster (C1), the chromatographic peak areas did not exceed the value of 200. For objects from the second, third, and fourth clusters (C2, C3, and C4), the peak areas reached maximum values of approximately 800, 1800, and 5000, respectively. Therefore, it can be observed that along with the increase in the apple juice content, the chromatographic peak areas also increased. On this basis, it can be concluded that these substances are characteristic of the volatile fraction of apples. In the samples classified to C5, an additional compound was detected which was 2-butanol, and the peak areas achieved value almost 9000. In the volatile fraction of the juices from the sixth cluster (C6), as many as nine of ten selected substances were detected. In these samples, the presence of 2-methylbutanol and *m*-xylene can be observed. 2-Methylbutanol is the alcohol identified in many apple varieties [36]. In contrast, *m*-xylene was detected in the headspace of apple bearing twig with leaves by Vallat et al. [38]. The presence of this compound may be caused by air pollution deposited on the surface of the fruit or residues of pesticides [43]. However, on the basis of the obtained results, it can be concluded that their concentration in the headspace of apple juice samples is small, as they were detected only in samples of 100% apple juice. In summary, the use of electronic nose in combination with the HCA method allowed the grouping of objects up to six clusters. However, it was impossible to distinguish samples of 100% orange juice (marked as 0.0) from samples adulterated with a 1% addition of apple juice (1.0).

To determine the quality of juice samples with greater precision, supervised statistical methods are used. Data obtained using the Heracles II apparatus were analyzed using several supervised algorithms to determine the use of which the more reliable results will be obtained. The effectiveness of these algorithms was evaluated using tenfold cross validation, and the results are given in Table 2.

**Table 2:** Cross validation of supervised algorithms used for classification of data from the analysis of fruit juice samples

Method	AUC	CA	Precision	Recall
RF	1.000	1.000	1.000	1.000
NB	0.943	0.675	1.000	0.200
NN	0.857	0.762	0.667	0.200
CT	0.950	0.938	1.000	0.900

*RF* random forrest classification,* NB* naïve bayes,* NN* neutral network,* CT* classification tree,* AUC* area under curve,* CA* accuracy, precision, recall (sensitivity)

The most reliable results were obtained for the Random Forest (RF) classification algorithm. It can be observed that the results of the evaluation of this algorithm regarding accuracy, precision, and sensitivity are 1.0. These results provide 100% effectiveness in predicting the Random Forest algorithm.

Random Forest is a kind of forecasting tools. This algorithm is a combination of decision trees [44]. Using this method, a very precise classification of the tested samples can be obtained. In addition, they are characterized by high accuracy, resistance to noise, simplicity, and speed of action [45]. These properties make Random Forest a useful tool for classifying objects even with a huge number of features. Based on the information obtained, it was decided to use this algorithm for further research.

The Random Forest method was trained using 67% randomly selected data to avoid error. The remaining data were used to test the chosen method. To present the prediction results for the selected supervised algorithm, Table 3 contains information about the sample confusion matrix for the Random Forest classifier. This matrix compares the percentage values of the proportions of real samples that were well classified. While sampling data for classification, 100% correct classification based on the degree of adulteration of orange juice has been repeatedly obtained.

**Table 3:** Confusion matrices of fruit juice samples classification using RF; scores are given as a proportion of predicted

Actual	Predicted								
	0.0	1.0	3.0	5.0	10.0	30.0	50.0	100.0	Σ
0.0	**100%**	0%	0%	0%	0%	0%	0%	0%	5
1.0	0%	**100%**	0%	0%	0%	0%	0%	0%	4
3.0	0%	0%	**100%**	0%	0%	0%	0%	0%	2
5.0	0%	0%	0%	**100%**	0%	0%	0%	0%	3
10.0	0%	0%	0%	0%	**100%**	0%	0%	0%	5
30.0	0%	0%	0%	0%	0%	**100%**	0%	0%	1
50.0	0%	0%	0%	0%	0%	0%	**100%**	0%	2
100.0	0%	0%	0%	0%	0%	0%	0%	**100%**	4
Σ	5	4	2	3	5	1	2	4	26

## Conclusion

The use of e-nose based on ultra-fast gas chromatography equipped with unsupervised and supervised chemometric methods is an effective tool for authentication fruit juice samples. This technique allows to omit sample preparation step and provides a low time-consuming single analysis. Based on the obtained results, it can be concluded that using HCA methods allowed to classify orange juice samples for unadulterated and adulterated with apple juice. Unfortunately, samples of orange juice containing 1.0% of apple juice were assigned to the group of unadulterated samples. More reliable results were achieved through the use of supervised statistical methods. The combination of e-nose measurements with Random Forest classifier made it possible to distinguish between particular orange juice samples based on the added volume of apple juice. The obtained results are the basis for further investigations. In the near future, the focus should be placed on the developed methodology, in which samples of fruit nectars and juices from concentrate will be considered. They are one of the most falsified ones.

## Experimental

### Sample preparation

Fruit juices were obtained at local distribution centres in Gdansk. Samples were NFC juices, i.e., orange juice, apple juice, and mixtures of orange and apple juice (1/3/5/10/30/50% v/v addition of apple juice). The juice mixtures were prepared immediately after their purchase. A sample of 5.0 ± 0.1 g of each fruit juice was poured into 20 cm^3^ glass vials that were then sealed with a cap with a silicone–PTFE membrane. Samples were stored for 24 h at 4 °C. For each type of samples, the analyses were performed in ten replicates.

### Instrumentation

The measurements were performed using an ultra-fast gas chromatograph Heracles II (Alpha MOS, Toulouse, France) equipped with a split/splitless injector and two flame ionization detectors (μFIDs). The two parallel linked capillary chromatographic columns used for separation were non-polar MXT-5 (diphenyl dimethylpolysiloxane, 10 m × 0.18 mm × 0.40 μm) and medium-polar MXT-1701 (cyanopropylphenyl polysiloxane, 10 m × 0.18 mm × 0.40 μm). Before the headspace analysis, samples have been incubated in 40 °C by 120 s and with agitation speed 500 rpm. The samples were injected by the HS 100 autosampler (Gerstel, Mülheim, Germany) with a 5.0 cm^3^ syringe and the injection volume was 2.5 cm^3^. The temperature of the injector and the detector were, respectively, at 200 and 270 °C. Hydrogen was used as carrier gas and its flow was kept constant at 250 mm^3^/s. The column temperature programming started at 40 °C, held for 5 s, and raised at a rate of 4 °C/s to 270 °C, maintained for the 30 s. The AlphaSoft 12.4 software was used to process the data. The aroma descriptors were obtained through the use of data collected from the AroChemBase.

### Data processing

Data from the e-nose measurements were exported and further processed using statistical methods. The chemometric analysis was performed using the Orange Canvas Data Mining v. 3.3.9 software (Bioinformatics Lab, University of Ljubljana, Slovenia). To normalise the features, they were centred by mean and scaled by the standard deviation. Ten chemical compounds were then selected based on one-way analysis of variance (ANOVA). The chromatographic peak areas corresponding to the selected chemical compounds were used as input data for hierarchical cluster analysis (HCA). Ward’s linkage method was applied. Clusters were created after cutting the dendrogram at a value corresponding to 3.0% of the maximum distance. In this way, six clusters were formed. The supervised statistical methods Random Forest (RF), Naïve Bayes (NB), Neural Network (NN), and Classification Tree (CT) were also used. RF method was chosen as a classifier based on the results of stratified tenfold cross validation. All the classifiers were taken with their optimal settings.
